# Mitochondrial Sco proteins are involved in oxidative stress defense

**DOI:** 10.1016/j.redox.2018.101079

**Published:** 2018-12-12

**Authors:** Aslihan Ekim Kocabey, Luise Kost, Maria Gehlhar, Gerhard Rödel, Uta Gey

**Affiliations:** Institute of Genetics, Technische Universität Dresden, 01062 Dresden, Germany

**Keywords:** SCO, synthesis of cytochrome *c* oxidase, SOD, superoxide dismutase, COX, cytochrome *c* oxidase, WT, wild type, PQ, paraquat, MD, menadione, L-AA, L-ascorbic acid, Sco proteins, Sod1, *Saccharomyces cerevisiae*, Oxidative stress response, ROS, Mitochondria

## Abstract

Members of the evolutionary conserved Sco protein family have been intensively studied regarding their role in the assembly of the mitochondrial cytochrome *c* oxidase. However, experimental and structural data, specifically the presence of a thioredoxin-like fold, suggest that Sco proteins may also play a role in redox homeostasis.

In our study, we addressed this putative function of Sco proteins using *Saccharomyces cerevisiae* as a model system. Like many eukaryotes, this yeast possesses two *SCO* homologs (*SCO1* and *SCO2*). Mutants bearing a deletion of either of the two genes are not affected in their growth under oxidative stress. However, the concomitant deletion of the *SOD1* gene encoding the superoxide dismutase 1 resulted in a distinct phenotype: double deletion strains lacking *SCO1* or *SCO2* and *SOD1* are highly sensitive to oxidative stress and show dramatically increased ROS levels.

The respiratory competent double deletion strain Δ*sco2*Δ*sod1* paved the way to investigate the putative antioxidant function of *SCO* homologs apart from their role in respiration by complementation analysis. Sco homologs from *Drosophila*, *Arabidopsis*, human and two other yeast species were integrated into the genome of the double deletion mutant and the transformants were analyzed for their growth under oxidative stress. Interestingly, all homologs except for *Kluyveromyces lactis* K07152 and *Arabidopsis thaliana* HCC1 were able to complement the phenotype, indicating their role in oxidative stress defense. We further applied this complementation-based system to investigate whether pathogenic point mutations affect the putative antioxidant role of hSco2. Surprisingly, all of the mutant alleles failed to restore the ROS-sensitivity of the Δ*sco2*Δ*sod1* strain.

In conclusion, our data not only provide clear evidence for the function of Sco proteins in oxidative stress defense but also offer a valuable tool to investigate this role for other homologous proteins.

## Highlights

• Concomitant deletion of *SCO* and *SOD1* leads to a high ROS sensitivity.
• SCO homologs from higher organisms can rescue the oxidative stress sensitive phenotype of the double deletion mutant.
• Pathogenic human Sco2 mutations affect the antioxidant function of the protein.
• The role of the Sco proteins in oxidative stress defense is discussed.

## Graphical abstract

**Figure:**
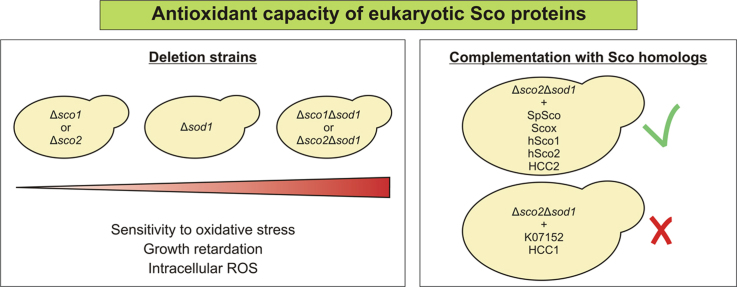

## 1 Introduction

Sco (synthesis of cytochrome *c* oxidase) proteins exist in almost all kinds of organisms ranging from simple prokaryotes to complex eukaryotes. The number of *SCO* genes varies among organisms: while prokaryotes possess up to seven [1], some eukaryotes harbor only one and most carry two *SCO* genes, probably as a result of a genome duplication process [2], [3].

Sco proteins were first identified in the yeast *Saccharomyces (S.) cerevisiae* as an essential component for the biogenesis of the cytochrome *c* oxidase (COX), the terminal enzyme complex of the mitochondrial respiratory chain [4]. Yeast Sco1 (ySco1) is essential for the assembly of COX [5], and its copper-binding properties [6] and physical interaction with yCox2 (cytochrome *c* oxidase subunit 2) suggests its role in delivering copper to the Cu_A_ center of yCox2 [7], [8]. Albeit the second Sco protein in yeast, ySco2, shows a high similarity to its paralog regarding amino acid sequence (71%) and structural features (thioredoxin-like domain) [9], only the deletion of *SCO1* results in a respiratory deficient phenotype, whereas the Δ*sco2* strain does not exhibit any obvious phenotype [10]. In contrast, human COX requires both Sco proteins (hSco1 and hSco2) for its assembly [11] and investigations on their structure and copper-binding ability [12], [13], [14] strengthened the proposed role in copper delivery to COX. However, further investigations on the function of the two human Sco proteins revealed distinct modes of action: while hSco1 most likely directly transfers copper to COX, hSco2 rather functions in activation of hSco1 by oxidizing its copper-coordinating cysteines [15]. Taking the results of studies in different organisms into consideration, the role of Sco proteins during COX assembly might vary and possible alternative functions were proposed [2], [16]. Especially the existence of several prokaryotes possessing *SCO* genes but no *COX2* and vice versa [1] even hint at distinct function(s) not related to COX biogenesis.

Interestingly, sequence alignments [16] as well as subsequent structural characterizations [12], [13], [17], [18] have revealed a thioredoxin-like fold as a structural homology between Sco proteins and antioxidant enzymes, such as peroxiredoxins and thiol-disulfide oxidoreductases. These findings are consistent with experimental results that demonstrated the broad roles of prokaryotic Sco proteins in the defense against oxidative stress and their function as disulfide reductases [19], [20], [21]. Studies on immortalized cells derived from patients who carry pathogenic mutations in the h*SCO1* gene (associated with hepatic failure and ketoacidotic coma [22]) and h*SCO2* (associated with fatal infantile cardioencephalomyopathy [23], [24], [25], [26], myopia 6 [27], [28], and leigh syndrome [23], [29]) suggest additional roles for the human homologs in copper homeostasis including thioredoxin activity and redox signaling [15], [30], [31].

Considering the functional and structural conservation among distant organisms and the available data on the diverse roles of Sco proteins, the question arises whether eukaryotic Sco proteins are also involved in oxidative stress defense as proposed for their prokaryotic counterparts. Due to the intertwining of mitochondrial respiration and the generation of reactive oxygen species (ROS) during the electron transport through the respiratory chain [32], we chose the facultative aerobic *S. cerevisiae* as a well-suited model organism [33], [34] to analyze the putative ROS defensive role of Sco proteins. In our approach, we investigated the phenotypes of strains lacking one of the two *SCO* genes (Δ*sco1* or Δ*sco2*) concomitant with another gene involved in redox homeostasis. Our study revealed that double deletion mutants lacking either *SCO1* or *SCO2* and the superoxide-dismutase *SOD1* (Δ*sco1*Δ*sod1* and Δ*sco2*Δ*sod1*) exhibit a pronounced sensitivity to oxidative stress associated with high intracellular ROS levels. These data not only provide strong evidence for a function of ySco proteins in redox balance. They also paved the way to easily analyze a ROS defensive function of Sco homologs from different eukaryotic organisms as well as pathogenic human *sco* mutant alleles for their ability to complement the oxidative stress sensitive phenotype of the Δ*sco2*Δ*sod1* strain.

## 2 Materials and methods

### 2.1 Bioinformatic analysis

The protein sequences were retrieved from the UniProt database [35] and pairwise alignments as well as calculation of similarity rates were done by Emboss Needle [36]. Mitochondrial targeting sequences were predicted with the MitoFates tool [37]. The transmembrane (TM) domain was predicted with TMpred [38] for SpSco and K07152; for the other Sco homologs, this information was retrieved either from literature or the UniProt database. The information regarding the thioredoxin-like domain was obtained from InterPro analysis [39].

### 2.2 Yeast strains, media and growth analysis

*S. cerevisiae* wild type (WT) strain BY4741 (Accession no. Y00000) and deletion strains Δ*sco1* (*SCO1*::kanMX4, Accession no. Y03174/Y13174), Δ*sco2* (*SCO2*::kanMX4, Accession no. Y03161/Y13161), Δ*sod1* (*SOD1*::kanMX4, Accession no. Y06913), Δ*sod2* (*SOD2*::kanMX4, Accession no. Y06605) and Δ*trx3* (*TRX3*::kanMX4, Accession no. Y07197/Y17197) were purchased from Euroscarf (Frankfurt, Germany). The *rho*^*0*^-strain KL14-4a [40] was used as a control strain lacking mitochondrial DNA. Double deletion strains were generated by crossing single deletion strains of opposite mating types, sporulation of the resulting diploids and subsequent dissection of single spore clones, which were genotypically characterized by PCR.

Yeast full media containing 2% glucose (YPD) and minimal media for selection of transformants were prepared as described [41] using media components from FORMEDIUM (Norfolk, UK). Paraquat (PQ), menadione (MD) and L-ascorbic acid (L-AA) (Sigma-Aldrich, St. Louis, MO) were added to YPD at the indicated concentrations.

For growth analysis on plates, cells were incubated in liquid YPD for 24 h followed by setting up a second culture (1:100) and incubation overnight. A dilution series from 10^4^ to 10^1^ cells was prepared of each strain and dropped onto the respective solid media. Plates were incubated at 30 °C for three days before growth was documented. Growth analysis in liquid media in 96-well plates was performed using the NEPHELOstar (BMG Labtech, Ortenberg, Germany) as described [42].

The viability of yeast cells was assessed using methylene blue staining. Cells were diluted 1:10 in 0.02% (w/v) methylene blue solution (pH 7.2) and incubated for 10 min at room temperature. The ratio of living (colorless) to dead (blue) cells was determined by counting cells in an improved Neubauer hemocytometer.

### 2.3 Generation of complementation constructs and site directed mutagenesis

To test the complementation with heterologous *SCO* genes, strains were generated by homologous recombination of the integration cassettes [43] into the *SCO2* chromosomal locus of the strain Δ*sco2*Δ*sod1*. Each cassette includes the gene of interest, a 3HA-tag for immunological detection and the *URA3* selection marker. The *SCO* genes were amplified using cDNA of the respective organism and the *3HA-URA3* cassette was PCR-amplified from the vector pUC19HA (kind gift of W. Zachariae, MPI-B Martinsried). A subsequent overlap-extension PCR combined both products using primers with overhangs for homologous integration. Point mutations in the *hSCO2* gene were introduced using mutagenic primers containing mismatches at the respective site. All primers used in this study and the generated recombinant and mutant strains are listed in Supplementary table S1 and S2, respectively. All constructs were approved by sequencing. Yeast transformation was performed according to Gietz and Woods [44].

### 2.4 Expression and localization analysis

For protein preparations, yeast cultures were grown overnight in 20 ml YPD and crude cytoplasmic and mitochondrial fractions were prepared as described previously [45]. Protein concentration (A_260_) was measured by NanoDrop (Thermo Fisher, Waltham, MA). Preparation of 15% SDS polyacrylamide gels and protein electrophoresis were carried out according to Laemmli [46]. For Western blot analysis, proteins were transferred onto a PVDF membrane (Millipore, Billerica, MA), probed with primary antibodies and detected with HRP-conjugated secondary antibodies using the ECL Prime Kit (GE Healthcare, Little Chalfont, UK). Primary antibodies were directed against HA (Roche, Basel, Switzerland) and Cox2p (Invitrogen, Carlsbad, CA), respectively.

### 2.5 ROS measurements

Yeast strains were inoculated in 5 ml of YPD (pre-culture), grown for 16 h and used to set up the main cultures (adjusted to an OD_600_ of 0.1 in fresh YPD). PQ was added after 4 h in a final concentration of 1 mM (DCF assay) or 0.1 mM (Amplex Red and lipid peroxidation assay). After 24 h treatment, OD_600_ of the samples was determined and ROS levels were quantified directly or indirectly by the different assays.

#### 2.5.1 DCF staining

Intracellular ROS was determined using 2′,7′-dichlorodihydrofluorescein diacetate (DCFH-DA, [47]). 10^7^ cells were harvested by centrifugation (3500×*g*, 5 min, RT), washed twice with 1x PBS, resuspended in 1 ml 1x PBS and diluted 1:10 before DCFH-DA was added to a final concentration of 20 µM. The cells were incubated for 4 h at 30 °C and then shortly sonicated before fluorescence intensity (ex: 488 nm, em: 527 ± 30 nm) of the cells was measured using flow cytometry (“CyFlow”, Partec, Görlitz, Germany).

#### 2.5.2 Amplex Red staining

The release of hydrogen peroxide from cells as an indicator for ROS was measured with the Amplex Red hydrogen peroxide/peroxidase assay kit (Invitrogen). Cells were harvested by centrifugation (3500×*g*, 5 min, RT), washed once with 1x PBS and subsequently incubated for 30 min in the presence of 10 μM Amplex Red and 0.2 U/ml of horseradish peroxidase. Cells were pelleted by centrifugation (3000×*g*, 20 s, RT), the supernatant was transferred to 96-well plates and the fluorescence (ex: 525 ± 10 nm, em: 585 ± 20 nm) was measured using the Infinite M200 plate reader (TECAN, Männedorf, Switzerland). A sample without cells was used as a blank and the amount of hydrogen peroxide was calculated from a standard curve with known concentrations.

#### 2.5.3 Lipid peroxidation assay

The Bioxytech^®^ LPO-586 kit (Hölzel Diagnostika, Cologne, Germany) was used to measure the lipid peroxide levels in cells. Cells were harvested by centrifugation (3500×*g*, 5 min, RT), washed once with ddH_2_O and resuspended in 500 µl of lysis buffer (1x PBS, 5 mM butylated hydroxytoluene). Then the cells were disrupted for 5 min in the presence of glass beads using the Mixer mill MM200 (Retsch, Haan, Germany). Subsequently, samples were centrifuged (3500×*g*, 5 min, 4 °C) and the supernatant was used to determine the protein concentration (A_260_) by NanoDrop (Thermo Fisher, Waltham, MA). Lipid peroxidation levels were calculated according to the manufacturer´s instructions. Hydrochloric acid was utilized in all experiments to specifically measure the malondialdehyde (MDA) amount in the samples. All samples were run in duplicates, and the absorbance at 586 nm was measured with the Infinite M200 plate reader. A separate blank was prepared for each sample according to the manufacturer's instructions and MDA levels were normalized to the protein concentrations.

### 2.6 SOD and COX activity measurements

For the measurement of enzyme activities, mitochondria were enzymatically prepared. To this end, yeast cells were grown in YPD in baffled flasks and mitochondria were isolated and purified by single gradient centrifugation as described by Meisinger et al. [48]. An EDTA-free protease inhibitor cocktail (Roche) and 1 mM 4-(2-aminoethyl) benzenesulfonyl fluoride (AppliChem, Darmstadt, Germany) were added throughout the preparations in order to prevent protein degradation.

SOD activity was assessed using the “SOD determination kit 19160” (Sigma Aldrich) following the manufacturer's instructions. This system relies on the inhibition of the reduction of a formazan dye derivate by SOD activity. The photometric measurement was performed in triplicates in 96-well plates in a volume of 200 µl containing 10 µg protein for each sample. Reactions were incubated at 30 °C and followed by measuring the absorption at 450 nm using the Infinite M200 plate reader. The SOD activity (% inhibition) was calculated from the final absorption value after 20 min incubation using the formula provided in the manual.

COX activity was determined following the oxidation of reduced cytochrome *c* by absorption measurement. Cytochrome *c* (from *S. cerevisiae*, Sigma-Aldrich) was reduced by the addition of sodium sulphite and purified using an Amicon^®^ Ultra 0.5 Centrifugal Filter column (Merck Millipore). The photometric measurement was performed in triplicates in 96-well plates in a volume of 100 µl containing 120 µM of reduced cytochrome *c* and 10 µg of mitochondria for each sample. The rate of oxidation of cytochrome *c* (delta E) was determined at 30 °C by following the decrease in absorption at 548 nm using the Infinite M200 plate reader. After 5 min, 5 µl 10 mM potassium hexacyanoferrate (III) (K_3_[Fe(CN)_6_]) was added to oxidize all remaining cytochrome *c*. The rate constant k, which is indirectly proportional to COX activity, was calculated with the formula: k = ln (delta E_0_/delta E_1_). Hereby delta E_0_ represents the difference between the absorption value at the reaction start and at the end, while delta E_1_ is the difference between the absorption at the start and after 1 min.

### 2.7 Statistical analysis

All data are shown as the mean ± standard deviation. The significance of differences between samples was evaluated by using two-tailed *t*-test. Analyses were done with GraphPad Prism 5 software (GraphPad Software, San Diego, California).

## 3 Results

### 3.1 Concomitant deletion of either *SCO1 or SCO2 and SOD1* causes a high sensitivity to oxidative stress

**Fig. 1:**
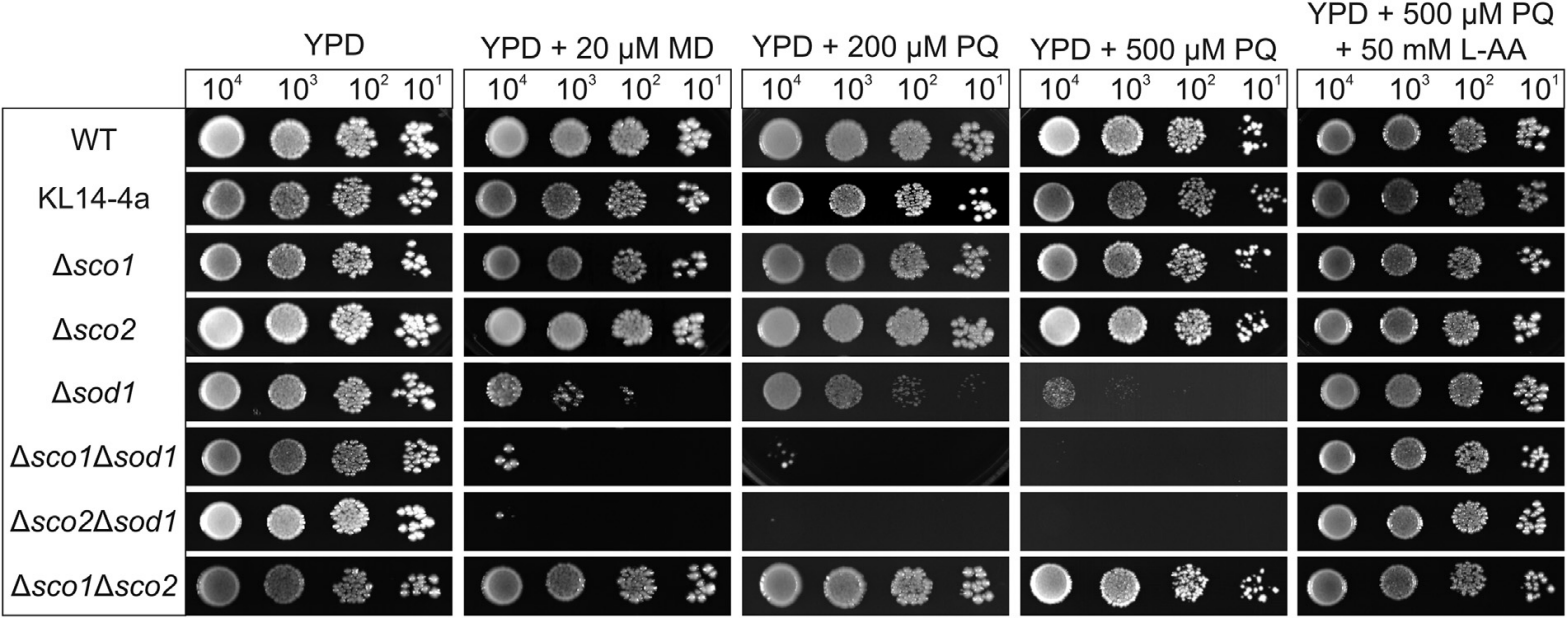
**Growth analysis under oxidative stress**. Cells of wild type (WT), the *rho*^*0*^-strain KL14-4a and the indicated single and double deletion strains were dropped in a dilution series (10^4^–10^1^ cells) onto YPD plates with the indicated concentrations of menadione (MD), paraquat (PQ) and L- ascorbic acid (L-AA). Growth was documented after incubation at 30 °C for three days.

The presence of a thioredoxin-like fold may hint at a role of the Sco proteins in redox balance or oxidative stress defense. The lack of proteins involved in these processes often lead to an increased sensitivity of yeast cells to oxidative stress and hence to growth retardation [49]. However, neither the single deletion of one of the *SCO* genes (Δ*sco1* or Δ*sco2*) nor the double deletion of both (Δ*sco1*Δ*sco2*) led to a diminished growth of the yeast strains on media containing ROS-inducing agents like menadione and paraquat (Fig. 1).

Possibly other proteins with overlapping functions are able to compensate for the absence of the respective Sco protein function. Hence, we generated different yeast strains with double deletions lacking one of the two Sco proteins and an enzyme with known function in oxidative stress defense. Interestingly, a specific phenotype could be observed for the strains with concomitant deletion of *SCO1* or *SCO2* and the superoxide dismutase 1 (*SOD1*), which were highly sensitive to ROS inducing agents (Fig. 1). Both the *rho*^*0*^-strain KL14-4a lacking mitochondrial DNA and respiratory deficient *COX* mutants (Δ*cox7*, Δ*cox7*Δ*sod1*, Δ*cox17*, Δ*cox17*Δ*sod1*) (Fig. S1) showed no increased sensitivity to ROS inducing agents. This observation excludes that the growth retardation under oxidative stress may be due to a secondary effect of the respiratory deficiency of Δ*sco1* [10]. The presence of the antioxidant ascorbic acid (L-AA) counteracted the growth inhibition of the double deletion strains (Fig. 1). This result demonstrates that elevated ROS levels underlie the observed growth phenotype and sustains the hypothesis that Sco proteins contribute to cellular redox homeostasis. This idea is further supported by the finding that a double deletion strain lacking *SOD1* and *TRX3* encoding the mitochondrial thioredoxin (Δ*sod1*Δ*trx3*) exhibited a similar ROS sensitive phenotype (Fig. S1). Interestingly, only the concomitant deletion with *SOD1*, but not *SOD2* encoding the second yeast superoxide-dismutase, led to the additive growth retardation of ∆*sco1* or ∆*sco2* mutant strains (Fig. S1).

### 3.2 Oxidative stress leads to highly increased ROS levels in double deletion strains Δ*sco1*Δ*sod1* and Δ*sco2*Δ*sod1*

**Fig. 2:**
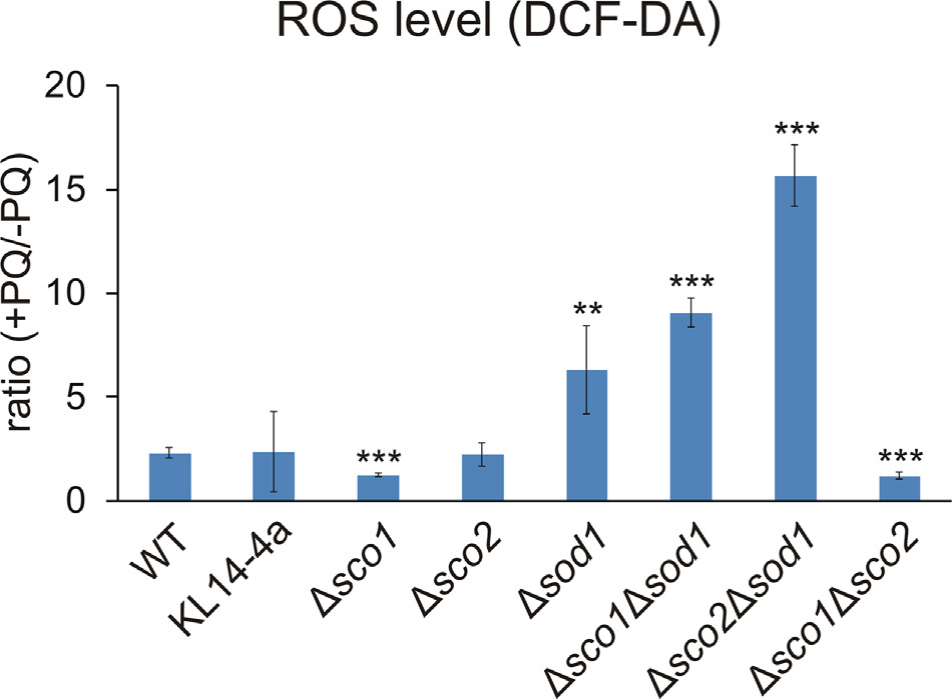
**Measurement of ROS levels in yeast*****SCO*****and*****SOD*****deletion mutants**. Wild type (WT), the *rho*^*0*^-strain KL14-4a and the indicated single and double deletion strains were grown in YPD with or without the addition of 1 mM PQ for 24 h, stained with DCF-DA and analyzed by flow cytometry. Mean fluorescence intensities (MFI) of the cell populations were measured and given values indicate the ratio of treated (+PQ) to untreated (-PQ) sample (± standard deviation). Data derive from four independent experiments. Mean values were compared with WT using the unpaired two-tailed *t*-test; ** p-value≤ 0.01, *** p-value≤ 0.001.

To sustain the hypothesis that oxidative stress confers the diminished growth of the mutant strains, we stained cells with the ROS sensitive fluorescent dye DCF-DA upon cultivation in the absence or presence of the external ROS inducing agent PQ. We determined the mean fluorescence intensities of the cells (Fig. S2), and calculated the ratio of treated to untreated samples (Fig. 2) for each strain as an indicator of ROS change under elevated stress.

The results show a notable correlation between the respective fluorescence ratios (Fig. 2) and the growth phenotype: while the ROS levels were only slightly increased (up to 2-fold) in most of the strains including the respiratory deficient ones (Δ*sco1*, Δ*sco1*Δ*sco2,* KL14-4a), it was elevated approximately 6-fold in Δ*sod1* after PQ treatment. Remarkably, in the double mutants Δ*sco1*Δ*sod1* and Δ*sco2*Δ*sod1* the increase was more than 9-fold. These results strongly suggest that higher ROS levels are accountable for the growth inhibition of the double deletion strains and argue in favor of a role of the Sco proteins in oxidative stress defense. Furthermore, these observations clearly illustrate that rather the absence of the antioxidant than the respiratory function of the Sco proteins causes the growth phenotype under stress.

To exclude that differences in the fluorescence intensity are caused by ROS-mediated cell death, we analyzed the viability of the yeast cells after the 24 h PQ treatment (Fig. S3). Methylene blue staining indicated a cell viability rate of almost 100% in the WT but also in all mutant strains (∆*sod1* and Δ*sco1*Δ*sod1* and Δ*sco2*Δ*sod1*) after PQ treatment (Fig. S3A). We further analyzed the growth behavior of the PQ-stressed cells in fresh YPD medium. Although the lag-phase was slightly prolonged in the deletion mutants, all strains resumed growth and showed exponential growth rates similar to the WT (Fig. S3B). Hence, we conclude that the PQ treatment does not lead to cell death but rather induces a reversible growth arrest.

### 3.3 The concomitant deletions of *SOD1* and *SCO* genes do not have an additive effect on SOD and COX activities compared to the single deletion mutants

To assess the influence of the concomitant deletions of *SCO* genes and *SOD1* on superoxide conversion (SOD) or COX assembly (SCO), we measured the corresponding enzymatic activities in the respective yeast strains. The overall SOD activity is composed of both the Cu/Zn-SOD (ySod1) and the Mn-SOD (ySod2). While ySod2 is present in the mitochondrial matrix, ySod1 is mainly localized in the cytoplasm with a small portion (~1–5%) residing in the mitochondrial intermembrane space [50]. Cells were subfractionated in order to differentiate cytosolic and mitochondrial SOD activity.

**Fig. 3:**
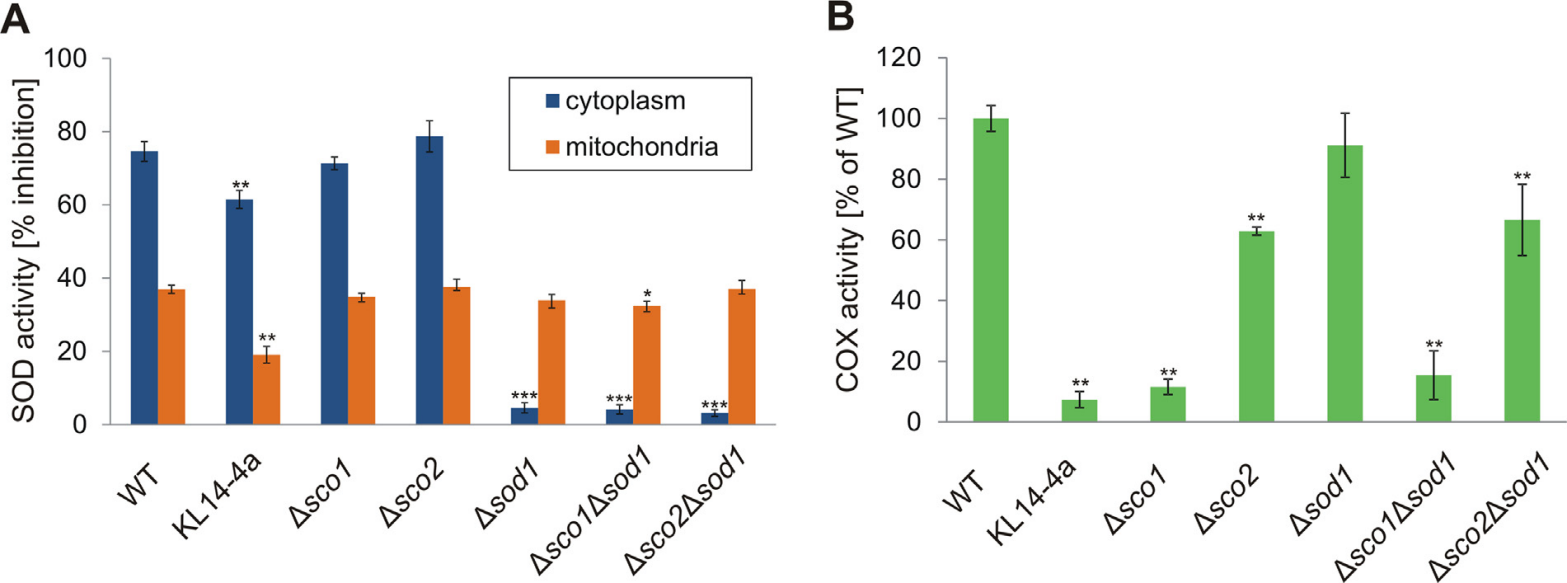
**SOD (A) and COX (B) activity in*****SCO*****and*****SOD*****single and double deletion mutants. A.** Mitochondria and cytoplasmic fractions were prepared and SOD activity was measured and calculated as described in the material and methods section. Mean values derive from three independent measurements (± standard deviation). **B.** COX activity was determined in purified mitochondria by measuring the conversion of reduced cytochrome *c* to its oxidized form. Mean values derive from triplicates of three independent experiments (± standard deviation). Values were compared with the respective WT sample using the unpaired two-tailed *t*-test; * p-value ≤ 0.05, ** p-value ≤ 0.01, *** p-value ≤ 0.001.

As expected cytosolic SOD activity could not be detected in *sod1* deletion strains, whereas it was not affected by *SCO* deletions (Fig. 3A). The mitochondrial activities – originating from the activity of ySod1 and ySod2 – were similar in all strains except for the *rho*^*0*^-strain KL14-4a. This strain showed about half of the WT activity, possibly due to the overall diminished metabolic activity in mitochondria. Mitochondrial SOD activities were only marginally diminished in the Δ*sod1* strains, reflecting the small portion of ySod1 in mitochondria. The concomitant deletion of either of the two *SCO* genes did not alter the SOD activity in comparison to the single deletion strain.

The measurements of the COX activities in purified mitochondria revealed the expected effect of *sco* deletions (Fig. 3B): similar to the *rho*^*0*^-control strain (KL14-4a), the respiratory deficient Δ*sco1* strain showed almost no COX activity. In contrast, the activity was only diminished to 60% in the Δ*sco2* strain reflecting the minor importance of ySco2 for COX assembly in yeast [9]. The concomitant deletion of *SOD1* in the *sco* deletion strains did not cause an additive effect on COX activity. Hence, an (additional) influence of ySod1 on COX activity can be excluded.

### 3.4 Most *SCO* homologs from different organisms can complement the antioxidant function of ySco2

The observation that the double deletion mutants Δ*sco1*Δ*sod1* and Δ*sco2*Δ*sod1* are hypersensitive to oxidative stress not only strengthens the hypothesis of an antioxidant role of the Sco proteins but also paved the way for complementation analyses.

**Fig. 4:**
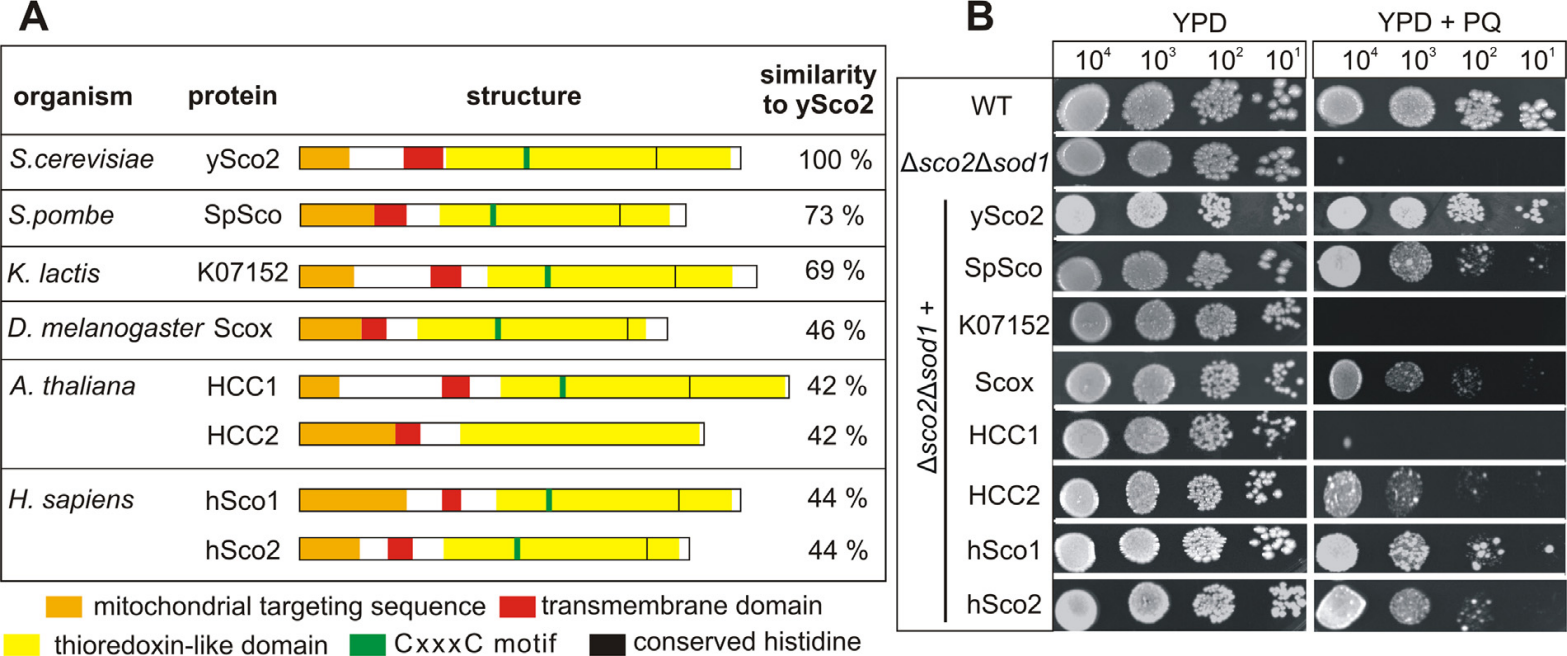
**Complementation analysis with*****SCO*****homologs from different organisms. A.** Overview of the analyzed Sco homologs: their structural features and sequence similarity to ySco2 are depicted. The positions of the mitochondrial target sequence (orange), transmembrane domain (red), thioredoxin-like domain (yellow), putative copper-binding motif CxxxC (green) and conserved histidine residue (black) are highlighted. **B.** Cells of the WT, the Δ*sco2*Δ*sod1* mutant strain and its transformed derivatives expressing the indicated Sco homologs were dropped in a dilution series (10^4^ to 10^1^ cells) onto YPD plates with or without 100 µM PQ. Growth was documented after incubation at 30 °C for three days.

To minimize the impact of respiratory chain assembly on redox homeostasis, the complementation assay was carried out in the respiratory competent strain Δ*sco2*Δ*sod1*. We selected homologs of organisms from different kingdoms and complexity: two yeast species (*Kluyveromyces* (*K.*) *lactis* K07152 and *Schizosaccharomyces* (*S.*) *pombe* SpSco), *Drosophila melanogaster* (Scox), *Arabidopsis thaliana* (HCC1 and HCC2) and *Homo sapiens* (hSco1 and hSco2). All homologs show a high sequence similarity to ySco2 (from 42% to 73%) and share a similar domain structure (Fig. 4A). They carry an amino-terminal mitochondrial targeting sequence, a single transmembrane domain, a thioredoxin-like domain and – with the exception of HCC2 – the amino acid motif important for copper binding (the conserved CxxxC motif and a C-terminally localized histidine residue at a distance of 84–87 amino acids).

All homologs were cloned from cDNAs of the respective organisms, C-terminally fused with an HA-tag and integrated into the former *SCO2* locus of the Δ*sco2*Δ*sod1* strain. The resulting strains were phenotypically characterized regarding their growth under oxidative stress (PQ). The homologs from *S. pombe* (SpSco), *Drosophila* (Scox), one of the *Arabidopsis* homologs (HCC2) and both human homologs (hSco1 and hSco2) were able to rescue the phenotype of the double deletion strain to some extent but less compared with ySco2 (Fig. 4B). In contrast, the *K. lactis* homolog K07152 and the *A. thaliana* homolog HCC1 failed to restore the antioxidant function of ySco2. To exclude that their inability to complement is caused by either lack of their expression or their mislocalization, we performed Western Blot analyses of cytoplasmic and mitochondrial protein fractions (Fig. S4). Both K07152 and HCC1 were detected in the mitochondrial fractions at the expected molecular weight (31 kDa and 34 kDa, respectively), although HCC1 has a weak expression. Hence, it is possible that their inability to complement the ySco2 function results from the low expression level (HCC1) and/or misfolding of these proteins. However, it is equally likely that the lack of complementation is due to structural and/or functional reasons.

### 3.5 ROS levels under oxidative stress reflect the antioxidant capacity of the different Sco homologs

**Fig. 5:**
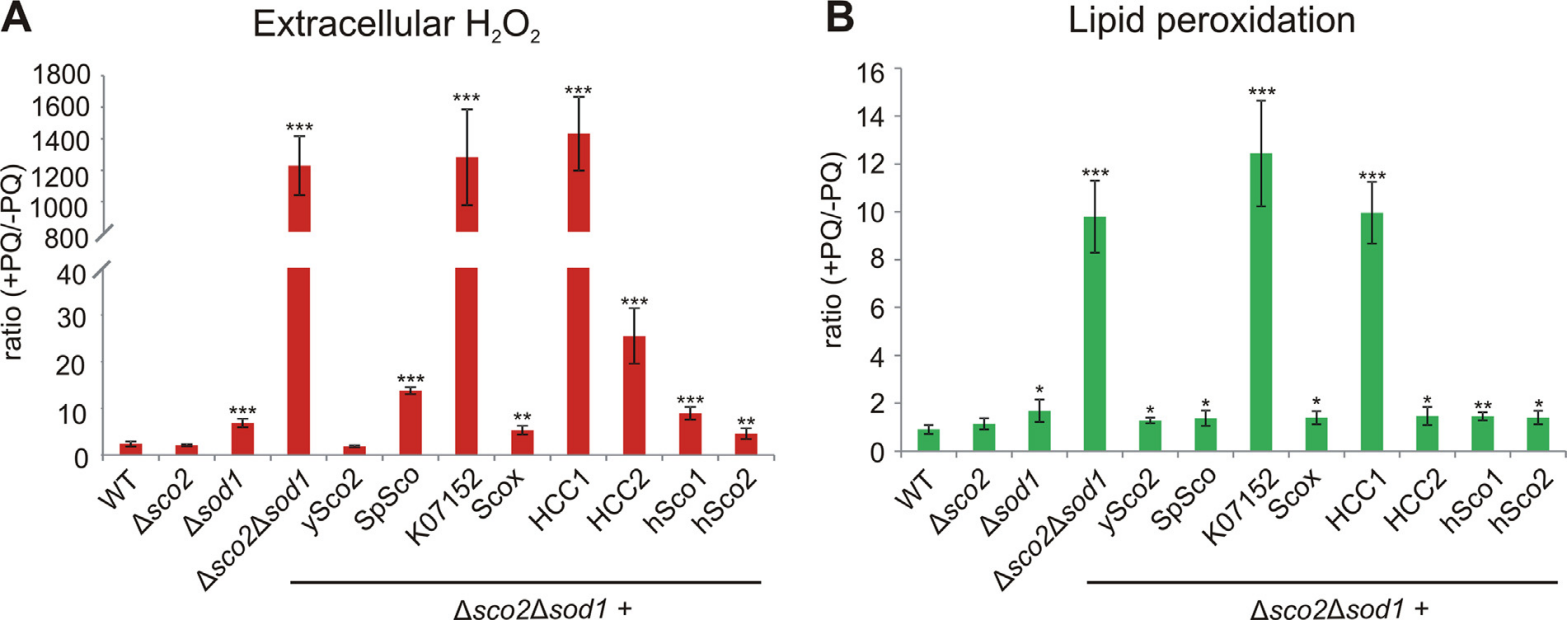
**Levels of extracellular H**_**2**_**O**_**2**_**(A) and lipid peroxidation levels (B) in deletion and recombinant yeast strains**. The indicated yeast strains were grown in YPD in the presence or absence of 100 µM PQ for 24 h. **A.** The cells were incubated with Amplex Red and the amount of hydrogen peroxide (µM H_2_O_2_/10^9^ cells) was calculated as described in the material and methods section. **B.** The malondialdehyde (MDA) concentration as an indicator for cellular lipid peroxidation was measured and normalized to the total protein amount (µM MDA/mg protein). Given data indicate the ratio of treated (+PQ) to untreated (-PQ) sample (± standard deviation) and are mean values from at least three independent experiments. Values were compared with WT using the unpaired two-tailed *t*-test; * p-value ≤ 0.05, ** p-value ≤ 0.01, *** p-value ≤ 0.001.

The ROS levels in the different strains were quantified directly (extracellular H_2_O_2_; Amplex Red; Fig. S5A) and indirectly (lipid peroxidation; Fig. S5B). To assess the change in ROS levels and ROS-mediated effects owing to elevated stress, we utilized the ratio of treated to untreated samples for each strain (Fig. 5).

The results obtained by both methods revealed the same trend: although all strains have higher ROS levels after PQ treatment compared with untreated ones, this increase is particularly apparent in Δ*sco2*Δ*sod1* and the transformants with the non-complementing homologs K07152 and HCC1. In contrast, a minor elevation was observed for Δ*sod1* as well as the strains harboring complementing *SCO* homologs.

The differences in ROS levels were especially pronounced in the Amplex Red staining (Fig. 5A) with more than 1000-fold increased H_2_O_2_ concentration in the Δ*sco2*Δ*sod1* and the non-complementing strains. This assay also revealed slight differences between the strains harboring complementing Sco homologs: SpSco, HCC2 and hSco1 showed slightly increased ROS ratios compared with the control, indicating a less efficient complementation than the authentic ySco2 protein.

The results of both ROS measurements corroborate the hypothesis of an antioxidant role of the complementing homologs and nicely support the correlation between growth rate and oxidant levels.

### 3.6 Pathogenic hSco2 mutant proteins fail to complement the antioxidant function of ySco2

**Fig. 6:**
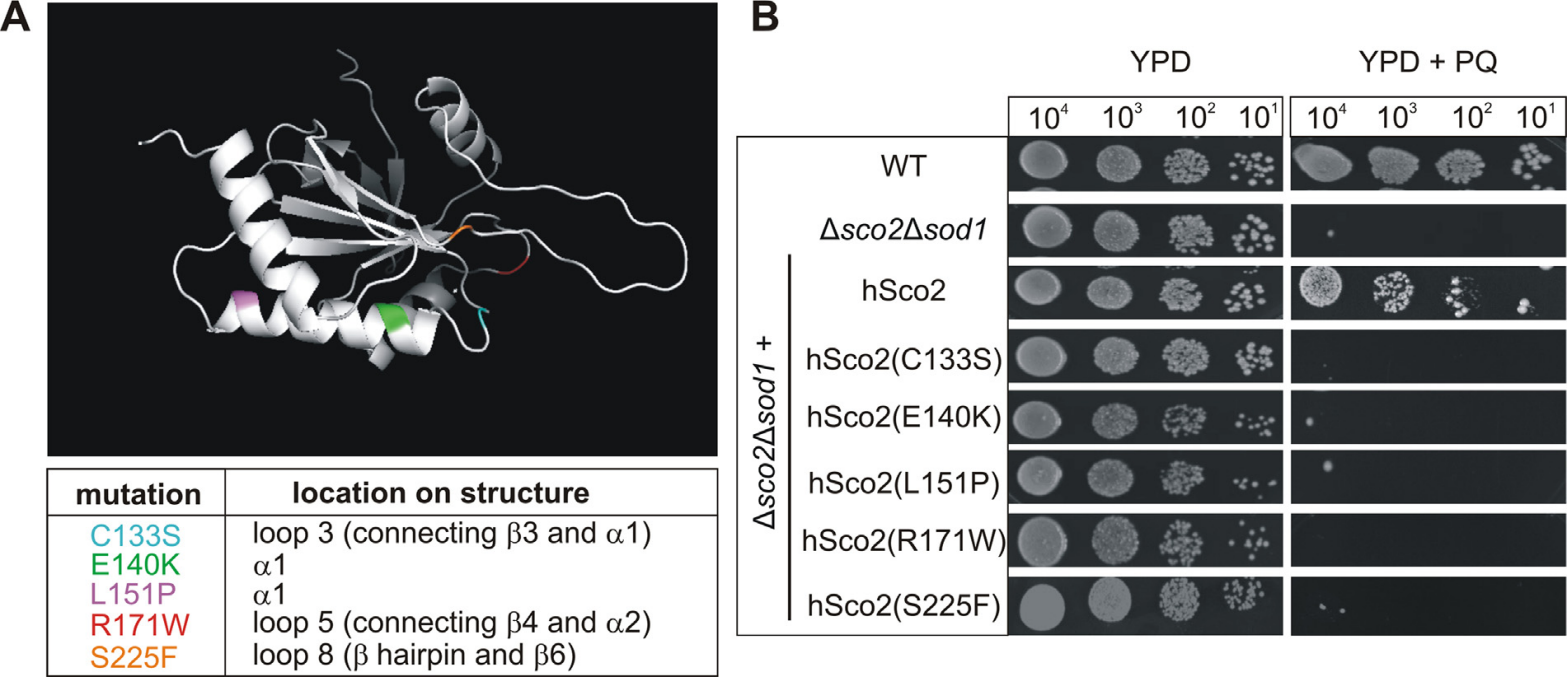
**Complementation assay with hSco2 variants harboring pathogenic point mutations. A.** Scheme of the location of selected pathogenic mutations in the structure of hSco2. The protein backbone of the soluble hSco2 [13] is given in grey. Mutations are shown at their exact positions on the protein structure and are highlighted in different colors. The figure was prepared with PyMOL by rearranging the available structure data retrieved from PDB (PDB: 2RLI). **B.** Cells of the wild type (WT), the Δ*sco2*Δ*sod1* mutant strain and its derivatives expressing different hSco2 variants were dropped in a dilution series (10^4^–10^1^ cells) onto YPD plates with or without 100 µM PQ. Growth was documented after incubation at 30 °C for three days.

The human homolog *hSCO2* was able to rescue the growth of the Δ*sco2*Δ*sod1* strain in the presence of PQ (Fig. 4). This observation provided the basis to assess the influence of pathogenic point mutations on the antioxidant property of the protein. Several pathogenic *hSCO2* mutations have been identified that lead to distinct diseases including fatal infantile cardioencephalomyopathy [25], [26], myopia 6 [28] and leigh syndrome [29]. We selected five pathogenic missense mutations (C133S, E140K, L151P, R171W, and S225F) for further analysis. The respective mutation sites are localized in different structural units (at either connecting loops or α-helices) of the thioredoxin-like domain that could be crucial for the putative redox function (Fig. 6A). Except for the C133S mutation, which is located in the conserved CxxxC motif, none of the mutations seems to be directly involved in copper binding [51].

The mutant genes were generated via site directed mutagenesis, integrated into the former *SCO2* locus in the Δ*sco2*Δ*sod1* strain and the growth of the respective transformants under oxidative stress was investigated (Fig. 6B).

Interestingly, all strains bearing mutant *hSCO2* alleles showed a normal growth on YPD under standard conditions but failed to grow under PQ-mediated oxidative stress. These results strongly argue that the disease-associated mutations affect the putative redox function of hSco2.

## 4 Discussion

Sco proteins are well characterized as copper chaperones that are important for COX assembly. In human, a crucial role of both Sco proteins for COX function has been shown [11], [15]. In contrast, in the yeast *S. cerevisiae* only the *sco1* deletion strain (∆*sco1*) exhibits respiratory deficiency due to a lack of COX activity [5], [7], while Δ*sco2* shows only a slightly reduced COX activity and no obvious phenotype (Fig. 3; [9]). Interestingly, the presence of a thioredoxin-like domain in the Sco protein structure as well as experimental data in prokaryotic [19], [21] and eukaryotic [52] species hint at the possibility that Sco proteins may also function in redox homeostasis. In this work, we provide strong experimental evidence for a role of Sco proteins in ROS defense by both phenotypic and biochemical analyses. The ∆*sco1* and ∆*sco2* strains with concomitant deletion of the superoxide dismutase gene *SOD1* show an increased sensitivity to ROS inducing agents, accompanied with high intracellular ROS levels. Moreover, the difference in ROS increase after oxidative stress between Δ*sco1*Δ*sod1* and Δ*sco2*Δ*sod1* strains may hint at a distinct antioxidant capacity of both Sco proteins under stress and point to their similar but not completely overlapping role in oxidative stress defense.

Interestingly, this ROS-sensitive phenotype can only be observed by double deletion of one of the *SCO* genes with *SOD1* but not *SOD2* (Fig. S1). This might be explained by the distinct localization of both enzymes: Sod2p is a mitochondrial matrix protein, while Sod1p is mainly localized in the cytoplasm with a small portion (approx. 1–5%) residing in the intermembrane space of mitochondria [50]. The fact that this submitochondrial compartment also harbors the catalytic C-terminal part of eukaryotic Sco proteins [10], [53] suggests a special importance of their activity in ROS defense when Sod1p is not present there. Possibly Sco proteins and Sod1p operate together in the mitochondrial intermembrane space to detoxify ROS or ROS-mediated products. Sco proteins might contribute to ROS defense via a thioredoxin-like function by reducing oxidized proteins. The similar phenotype of the double deletion strain lacking Sod1p and the mitochondrially localized thioredoxin Trx3p [54] (Δ*trx3*Δ*sod1*; Fig. S1) is in line with this idea.

The ROS-sensitive phenotype and high ROS levels of the double deletion strains not only support the hypothesis of an antioxidant function of the Sco proteins but also provided the opportunity to investigate this feature of Sco proteins of other eukaryotic organisms via complementation analysis. Interestingly, many but not all of the analyzed Sco homologs proved to functionally complement the phenotype of the Δ*sco2*Δ*sod1* deletion strain (Fig. 4). The complementation does not correlate with the extent of sequence homology, as the *K. lactis* homolog K07152, showing one of the highest sequence similarity to ySco2 with about 69%, was not able to rescue the phenotype. In the case of *Arabidopsis* homologs, only HCC2 was able to complement, while HCC1 was not. This result is in line with previous studies in plants that suggest an essential function of HCC1 in COX biogenesis but a stress defensive role for HCC2 [55], [56], [57]. Apparently, homologous Sco proteins – even within a single species – can possess divergent functions despite their high degree of sequence similarity.

Investigations on the homologs from higher organisms – particularly hSco1 [58], [59], [60], hSco2 [61], [62], [63] and the *Drosophila* homolog Scox [64], [65] – pointed to an interrelationship between these proteins and ROS but only in the context of respiration. Our assay overcame this limitation by the use of the respiratory competent strain Δ*sco2*Δ*sod1* that allowed us to test a possible antioxidant function of Sco proteins independent of their respiratory function. Although our data indicate a slight contribution of ySco2 to COX activity (Fig. 3), our complementation studies strongly suggest an independent antioxidant function for the majority of the analyzed homologs. The observation that the Δ*cox7*Δ*sod1* and Δ*cox17*Δ*sod1* strains, despite their impaired respiration, do not show an increased sensitivity to oxidative stress (Fig. S1) further strengthens the hypothesis of an antioxidant role not connected to the respiratory function of the Sco proteins. Moreover, reports on a role of Sco proteins in the oxidative stress defense in organisms like *Neisseria*, which do not harbor a COX [21], indicate that Sco functions related to COX assembly and ROS defense can be independently exerted. Taken together, the functions of members of the Sco protein family are apparently not uniform. While many Sco proteins are specifically involved in COX assembly, others are only important in ROS defense and some Sco proteins might act in both pathways.

The finding, that *hSCO2* is able to rescue the ROS-sensitivity of the ∆*sco2*∆*sod1* strain, allowed us to analyze the impact of known pathological *hSCO2* mutations on the antioxidant capacity. Former studies investigated the influence of the mutations by analyzing homologous mutations in the yeast counterparts [66] or *in vitro* with recombinant proteins [67]. We introduced five pathogenic *hSCO2* mutations (C133S, E140K, L151P, R171W, S225F) directly into the authentic *hSCO2* gene and investigated their effects in the complementation assay. All mutated alleles failed to complement the phenotype of the Δ*sco2*Δ*sod1* strain (see Fig. 6) and this nicely demonstrates the importance of the structural integrity of the thioredoxin-like fold of hSco2 to facilitate its antioxidant function.

Regarding the presence of a conserved copper-binding motif in the thioredoxin-like domain of almost all Sco proteins and their reported role in copper homeostasis [6], [14], [30], the question arises whether the antioxidant function may also depend on copper. However, the fact that the *Arabidopsis* homolog HCC2 is lacking the conserved copper-binding motif but able to confer oxidative stress tolerance to the ∆*sco2*∆*sod1* strain, argues against this idea. Additionally, other homologs harboring the CxxxC motif like K07152 and HCC1 were not able to rescue the stress sensitivity, suggesting that the domain structure itself rather than copper binding is important for this function. However, for ySco1 a copper coordination by alternative cysteines apart from the CxxxC motif has been shown [68]. As all Sco proteins including HCC2 contain additional cysteines, which could play a role in copper binding, a connection to copper metabolism cannot completely be excluded. Further studies are necessary to shed light on the role of copper in the functional properties of Sco proteins.

In summary, our data provide strong evidence that Sco proteins play a role in oxidative stress defense and redox homeostasis. Most likely, they are involved in ROS defense by reducing oxidized proteins *in vivo* via a thioredoxin-like function. Although some *in vitro* assays failed to show a thiol-oxidoreductase activity [17], [69], other studies provided evidence of such a function for prokaryotic Sco proteins [19] and hSco2 [15]. In the latter case, the oxidoreductase activity seems to be essential for loading copper to hSco1 and hence for COX assembly [30]. Some Sco proteins might also possess a broader substrate spectrum and perform a thioredoxin/oxidoreductase function on other (artificially oxidized) proteins, especially under oxidative stress. Another possibility could be a function of Sco proteins, either directly by ROS scavenging or indirectly by acting as a mitochondrial redox signaling molecule as suggested for the hSco1 protein [17]. The question whether the antioxidant function of Sco proteins is direct or indirect and the possible interconnected mechanisms behind this function remain to be elucidated.
