# Supplementary material for: Mitochondrial Sco proteins are involved in oxidative stress defense
Source: Redox Biol. 2018 Dec 12;21:101079. doi: 10.1016/j.redox.2018.101079 (PMC6307045; doi:10.1016/j.redox.2018.101079)
Supplement: Supplementary file 1 — Supplementary material. [file mmc1.docx]

**Supplementary material**

**Table S1: List of PCR primers used in this study.**

Overhang primers (#1 - #25; annealing regions are in bold) and overlap primers (final PCR step; #26 and #27) were used for amplification of the integration cassettes of the homolog *SCO* genes. Site directed mutagenesis primers (#28 - #37) were used for generation of the mutant hSCO2 variants.

| No | Name | Sequence (5´-3´) |
| --- | --- | --- |
| #1 | ySCO2 for | ACAGGATCTTTTATAATATCACCATAGAAAGGGGAATTTGGGAAGCGAGAGTAAA**ATGTTGAATAGTTCAAGAAA** |
| #2 | ySCO2 rev | **ATTGAAGATAAAAGAGTACC** |
| #3 | ySCO2 HA tag for | CTCTTTTATCTTCAAT**TCCGGTTCTGCTGCTAG** |
| #4 | ySCO2 rev | ACGCCCTTAAGCCCAATTAATAATTTGAACTGACAAGCATAATCATTTGACTTTC**CCTCGAGGCCAGAAGAC** |
| #5 | K07152 for | ACAGGATCTTTTATAATATCACCATAGAAAGGGGAATTTGGGAAGCGAGAGTAAA**ATGTTACGCAGCATTGTTC** |
| #6 | K07152 rev | **AAACAAGAAGGAATACCATTTGTC** |
| #7 | K07152 HA tag for | CAAATGGTATTCCTTCTTGTTT**TCCGGTTCTGCTGCTAG** |
| #8 | SpSCO1 for | ACAGGATCTTTTATAATATCACCATAGAAAGGGGAATTTGGGAAGCGAGAGTAAA**ATGTTTCGAAGGGGTTTAGTT** |
| #9 | SpSCO1 rev | **TTTCTGTTTCTTTCGGGACAA** |
| #10 | SpSCO1 HA tag for | CCGAAAGAAACAGAAA **TCCGGTTCTGCTGCTAG** |
| #11 | HCC1 for | ACAGGATCTTTTATAATATCACCATAGAAAGGGGAATTTGGGAAGCGAGAGTAAA**ATGGCGTCTGCTCTATGTAG** |
| #12 | HCC1 rev | **CTTCCGGTACTGACGGATC** |
| #13 | HCC1 HA tag for | GATCCGTCAGTACCGGAAG**TCCGGTTCTGCTGCTAG** |
| #14 | HCC2 for | ACAGGATCTTTTATAATATCACCATAGAAAGGGGAATTTGGGAAGCGAGAGTAAA**ATGCTTCCTTGTCGCCGT** |
| #15 | HCC2 rev | **CTGTGAAACAGAAGCAACTTC** |
| #16 | HCC2 HA tag for | TGCTTCTGTTTCACAG**TCCGGTTCTGCTGCTAG** |
| #17 | hSCO1 for | ACAGGATCTTTTATAATATCACCATAGAAAGGGGAATTTGGGAAGCGAGAGTAAA**ATGGCGATGCTGGTCCTAG** |
| #18 | hSCO1 rev | **GCTCTTTTTTCTGTATGGCC** |
| #19 | hSCO1 HA tag for | CCATACAGAAAAAAGAGC**TCCGGTTCTGCTGCTAG** |
| #20 | hSCO2 for | ACAGGATCTTTTATAATATCACCATAGAAAGGGGAATTTGGGAAGCGAGAGTAAA**ATGCTGCTGCTGACTCG** |
| #21 | hSCO2 rev | **AGACAGGACACTGCGGAA** |
| #22 | hSCO2 HA tag for | CCGCAGTGTCCTGTCT **TCCGGTTCTGCTGCTAG** |
| #23 | SCOX for | ACAGGATCTTTTATAATATCACCATAGAAAGGGGAATTTGGGAAGCGAGAGTAAA**ATGTCCCGCTCCCTGC** |
| #24 | SCOX rev | **GCTGAACCATCCCTTTTTG** |
| #25 | SCOX HA tag for | CAAAAAGGGATGGTTCAGC**TCCGGTTCTGCTGCTAG** |
| #26 | ov SCO2 for | ACAGGATCTTTTATAATATCACCATAG |
| #27 | ov SCO2 rev | ACGCCCTTAAGCCCAATT |
| #28 | hSCO2 C133S for | CACTCACAGCCCTGACATC |
| #29 | hSCO2 C133S rev | GATGTCAGGGCTGTGAGTG |
| #30 | hSCO2 E140K for | CCAGACAAGCTGGAGAAGCT |
| #31 | hSCO2 E140K rev | AGCTTCTCCAGCTTGTCTGG |
| #32 | hSCO2 L151P for | CAGCCGGAAGCAGAGC |
| #33 | hSCO2 L151P rev | GCTCTGCTTCCGGCTG |
| #34 | hSCO2 R171W for | CGAGTGGGACGACGTTG |
| #35 | hSCO2 R171W rev | CAACGTCGTCCCACTCG |
| #36 | hSCO2 S225F for | GACCACTTCATTGCCATCTAC |
| #37 | hSCO2 S225F rev | GTAGATGGCAATGAAGTGGTC |

**Table S2: Integration cassettes and primers used for the generation of the different recombinant/mutant strains.**

To generate the integration cassettes, two rounds of PCR were carried out. The primer sets used in the first round (standard PCR) are separated by semicolon. For the final PCR, overlap primers #26 and #27 were used for all constructs.

| **Recombinant/ mutant strain** | **Integration cassette** | **Primers for standard PCR** |
| --- | --- | --- |
| ySCO2 | *ySCO2*-3HA-*URA3* | #1, #2; #3, #4 |
| K07152 | *K07152*-3HA-*URA3* | #5, #6; #7, #4 |
| SpSCO1 | *SpSCO1*-3HA-*URA3* | #8, #9; #10, #4 |
| HCC1 | *HCC1*-3HA-*URA3* | #11, #12; #13, #4 |
| HCC2 | *HCC2*-3HA-*URA3* | #14, #15; #16, #4 |
| hSCO1 | *hSCO1*-3HA-*URA3* | #17, #18; #19, #4 |
| hSCO2 | *hSCO2*-3HA-*URA3* | #20, #21; #22, #4 |
| SCOX | *SCOX*-3HA-*URA3* | #23, #24; #25, #4 |
| hSCO2 (C133S) | *hSCO2* (C133S)-3HA-*URA3* | #26, #29; #27, #28 |
| hSCO2 (E140K) | *hSCO2* (E140K)-3HA-*URA3* | #26, #31; #27, #30 |
| hSCO2 (L151P) | *hSCO2* (L151P)-3HA-*URA3* | #26, #33; #27, #32 |
| hSCO2 (R171W) | *hSCO2* (R171W)-3HA-*URA3* | #26, #35; #27, #34 |
| hSCO2 (S225F) | *hSCO2* (S225F)-3HA-*URA3* | #26, #37; #27, #36 |

**

**

**Figure S1: Concomitant deletion of *SCO1/2* and *SOD2* does not result in a ROS-sensitive phenotype.** Cells of wild type (WT) and the indicated single and double deletion mutants were dropped in a dilution series onto YPD plates with or without 200 µM PQ. Growth was documented after incubation at 30 °C for three days.


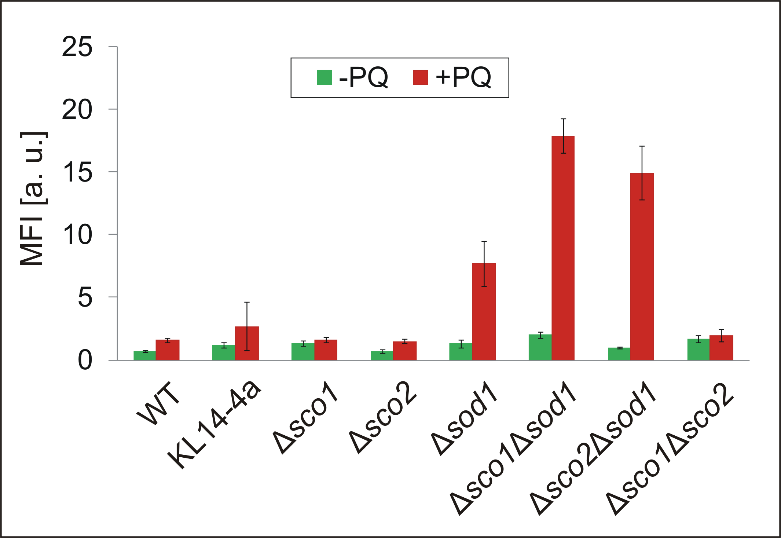


**Figure S2: Measurement of intracellular ROS levels in yeast *SCO* and *SOD* deletion mutants.** Wild type (WT), the *rho^0^*-strain KL14-4a and the indicated single and double deletion strains were grown in YPD with (red) or without (green) the addition of 1 mM PQ for 24 h, stained with DCF-DA and analysed by flow cytometry. Mean fluorescence intensities (MFI) of the cell populations derive from four independent experiments (+/- standard deviation).

**
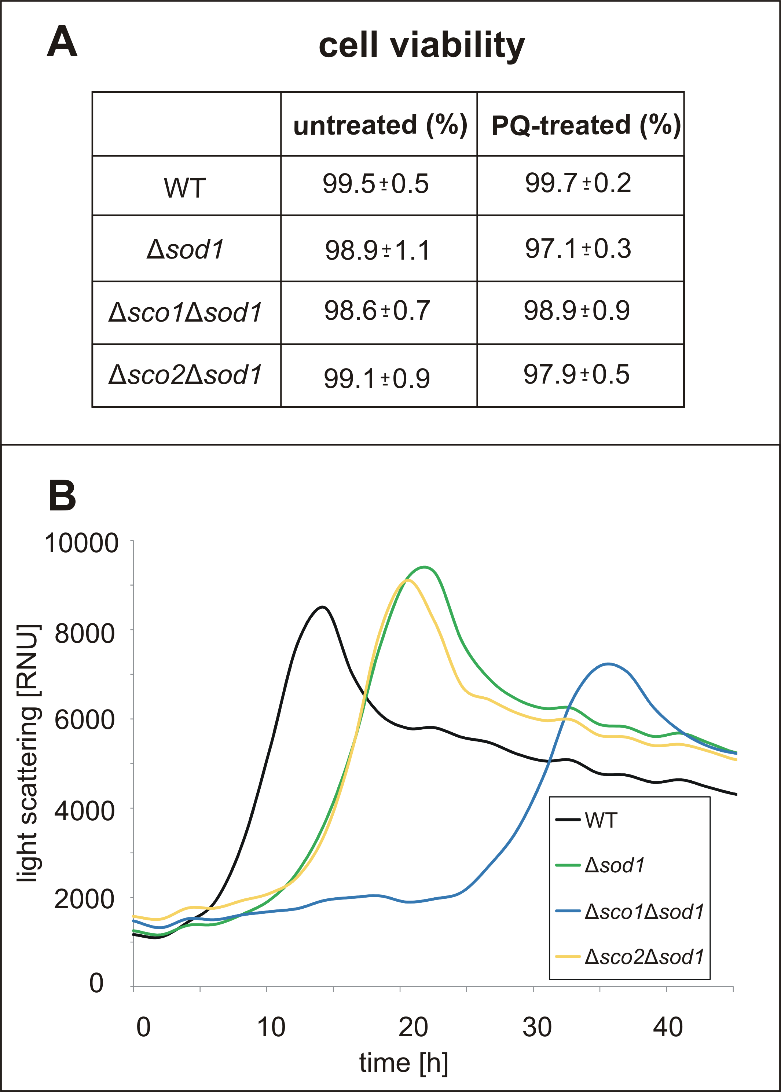
**

**Figure S3: Double deletion mutants are only growth arrested under oxidative stress and can recover in fresh YPD.** For the indicated yeast strains, cultures with an OD_600_ of 0.1 were set up and grown in YPD with the addition of 1 mM PQ for 24 h. **A.** The cell viability was determined by methylene blue staining (see methods for details). Given values are mean values from three independent experiments (+/- standard deviation). **B.** Cells stressed with PQ were used to set up new cultures in fresh YPD and growth behaviour was followed by measuring light scattering in with the nephelometer for 48 h.

**
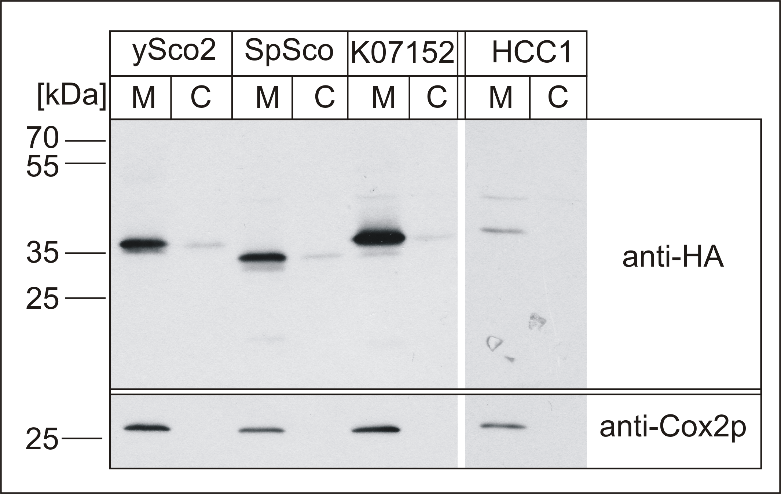
**

**Figure S4: Homologous Sco proteins are expressed and targeted to mitochondria in yeast.** Mitochondrial (M) and cytoplasmic (C) fractions were isolated from the Δ*sco*2Δ*sod1* strain expressing the indicated Sco homologs. 100 µg of protein per lane were loaded on a 15 % SDS-PAGE and Western Blot analysis was performed using antibodies directed against the HA-tag and Cox2p (used as mitochondrial marker).


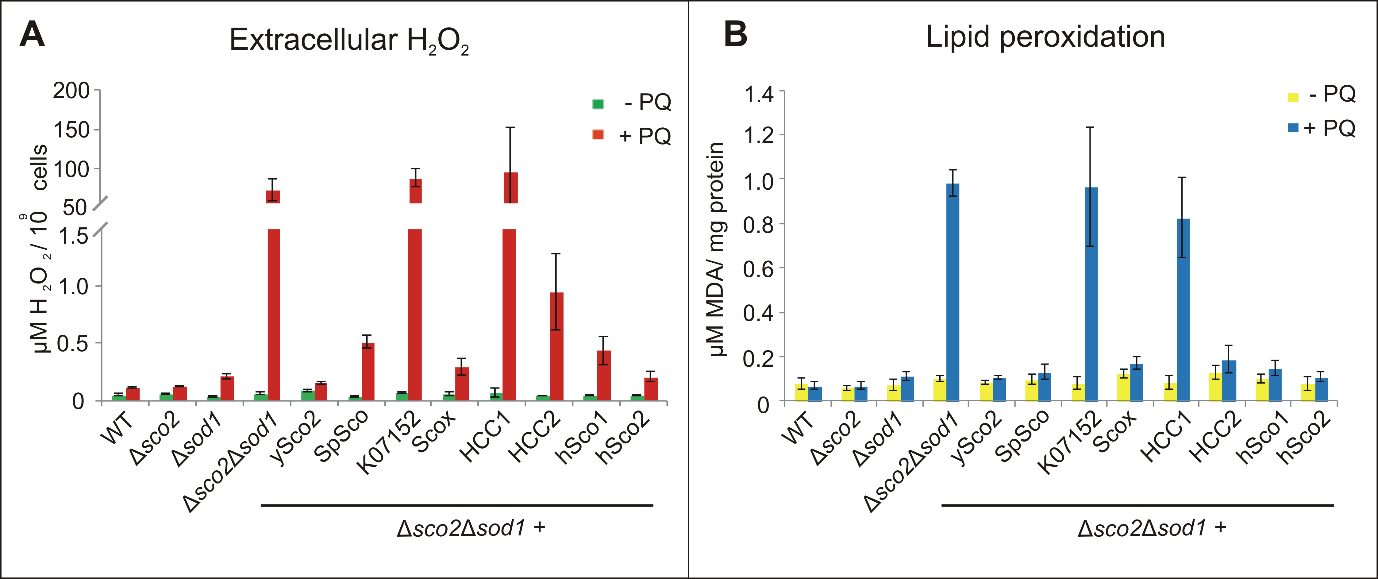


**Figure S5: Measurement of extracellular H_2_O_2_ (A) and lipid peroxidation levels (B) in deletion and recombinant yeast strains.** WT and the indicated deletion and recombinant yeast strains were grown in YPD with (red or blue) or without (green or yellow) the addition of 100 µM PQ for 24 h. **A.** The cells were incubated with Amplex Red and the amount of hydrogen peroxide was calculated as described in the material and methods section. **B.** The malondialdehyde (MDA) concentration as an indicator for cellular lipid peroxidation was measured and normalized to the total protein amount. All values are mean values from at least three independent experiments (+/- standard deviation).
